# Precise estimation of genomic regions controlling lodging resistance using a set of reciprocal chromosome segment substitution lines in rice

**DOI:** 10.1038/srep30572

**Published:** 2016-07-28

**Authors:** Taiichiro Ookawa, Ryo Aoba, Toshio Yamamoto, Tadamasa Ueda, Toshiyuki Takai, Shuichi Fukuoka, Tsuyu Ando, Shunsuke Adachi, Makoto Matsuoka, Takeshi Ebitani, Yoichiro Kato, Indria Wahyu Mulsanti, Masahiro Kishii, Matthew Reynolds, Francisco Piñera, Toshihisa Kotake, Shinji Kawasaki, Takashi Motobayashi, Tadashi Hirasawa

**Affiliations:** 1 Institute of Agriculture, Graduate School, Tokyo University of Agriculture and Technology, Fuchu, Tokyo 183-8509, Japan; 2 NARO Agrogenomics Research Center, Tsukuba, Ibaraki 305-8602, Japan; 3 Japan International Research Center for Agricultural Sciences, Tsukuba, Ibaraki 305-8686, Japan; 4 Bioscience and Biotechnology Center, Nagoya University, Nagoya, Aichi 464-8601, Japan; 5 Agricultural Research Institute, Toyama Agricultural, Forestry & Fisheries Research Center, Toyama, Toyama 939-8153, Japan; 6 International Rice Research Institute, Los Banos, Philippines; 7 International Maize and Wheat Improvement Center, Texcoco, 56237, Mexico; 8 Graduate School of Science and Engineering, Saitama University, Saitama 338-8570, Japan; 9 Department of Plant Physiology, National Institute of Agrobiological Sciences, Tsukuba, Ibaraki 305-8602, Japan

## Abstract

Severe lodging has occurred in many improved rice varieties after the recent strong typhoons in East and Southeast Asian countries. The *indica* variety Takanari possesses strong culm characteristics due to its large section modulus, which indicates culm thickness, whereas the *japonica* variety Koshihikari is subject to substantial bending stress due to its thick cortical fibre tissue. To detect quantitative trait loci (QTLs) for lodging resistance and to eliminate the effects of genetic background, we used reciprocal chromosome segment substitution lines (CSSLs) derived from a cross between Koshihikari and Takanari. The oppositional effects of QTLs for section modulus were confirmed in both genetic backgrounds on chromosomes 1, 5 and 6, suggesting that these QTLs are not affected by the genetic background and are controlled independently by a single factor. The candidate region of a QTL for section modulus included *SD1*. The section modulus of NIL-sd1 was lower than that of Koshihikari, whereas the section modulus of NIL-SD1 was higher than that of Takanari. This result indicated that those regions regulate the culm thickness. The reciprocal effects of the QTLs for cortical fibre tissue thickness were confirmed in both genetic backgrounds on chromosome 9 using CSSLs.

The successful breeding of rice for yield, which started in the 1960s, achieved an increase in grain production through the development of high-yielding and semi-dwarf rice varieties, thereby averting a predicted large-scale famine. This achievement is referred to as the Green Revolution^1^. One cause of the high grain production in the Green Revolution was the widespread use of chemical fertilizers for the cultivation of high-yielding and semi-dwarf varieties^2^,^3^. Under heavy fertilization, traditional rice varieties grow excessively tall and are susceptible to lodging, which bends entire culms or their basal parts and results in a significant loss of yield. By contrast, semi-dwarf varieties are resistant to lodging. Many improved semi-dwarf varieties have been developed and cultivated worldwide. However, despite the short stature conferred by the *semidwarf1* (*sd1*) gene, lodging occurs in many of these improved rice varieties when strong typhoons hit East and Southeast Asian countries. Furthermore, heavy yield losses due to lodging have been observed in other gramineous crops.

In an effort to avert a food crisis in the 21st century, rice breeders are attempting to develop varieties with increased plant biomass and harvest indexes to further increase the grain yield. Recently, several genes associated with grain yield and biomass production have been identified, including *GN1A*^4^,^5^, *GS3*^6^, *DEP1*^7^, *APO1*^8^, *WFP*^9^ and *NAL1*^10^. However, to sustain this breeding objective, it is also necessary to develop new strategies to improve lodging resistance in rice. Instead of relying solely on the *sd1* gene, alternative genes controlling culm strength can be utilized to develop improved rice varieties.

For lodging resistance, it is important that the physical strength of the basal culm is reflected in its bending moment at breaking (M). M is composed of a section modulus (SM), which indicates culm thickness, and the bending stress (BS), which indicates culm stiffness^11^. SM is associated with outer diameter and culm wall thickness. By contrast, BS is associated with properties such as the morphology of cortical fibre tissue^12^ and cell wall components such as lignin, cellulose and hemicellulose^13^. It is important to identify quantitative trait loci (QTLs) for those traits associated with culm strength because those are quantitative traits controlled by multiple genes^11^. We need to identify candidate genomic regions of those component traits by dissecting culm strength.

In our previous studies comparing traits associated with lodging resistance among rice varieties, Koshihikari was found to possess a small SM but a large BS^11^. Thick cortical fibre tissue and high densities of accumulated cell wall components such as cellulose and hemicellulose are responsible for the high BS in Koshihikari^14^,^15^. By contrast, Takanari has a large SM due to a large outer diameter and a small BS due to thin cortical fibre tissue.

It has been reported that cortical fibre tissues are very important for BS^12^. The superior lodging resistant variety Leaf Star has well-developed and thick cortical fibre tissues, which are inherited from the *japonica* variety Koshihikari, compared with the *tropical japonica* line Chugoku 117^14^. No QTLs associated with cortical fibre tissue thickness have been reported in rice or other gramineous crops, although it is necessary to identify the QTLs for these traits.

To identify and facilitate the genetic analysis of complex traits in rice, a series of chromosome segment substitution lines (CSSLs) and near-isogenic lines (NILs) have been developed^16^. The advantages of using CSSLs are the precise estimation of genomic regions for complex traits and the detection of minor QTLs^17^. In our previous study using Habataki CSSLs on the Koshihikari background, *ABERRANT PANICLE ORGANIZATION1* (*APO1*), which was previously reported to control panicle structure^8^, showed enhanced culm strength^18^. Therefore, CSSLs are a useful tool to estimate the genomic regions for quantitative traits associated with lodging resistance^18^. Recently, Takai *et al*.^19^ developed reciprocal CSSLs derived from a cross between Koshihikari and Takanari by repeated backcrossing, self-pollination and marker-assisted selection. They used these CSSLs to detect QTLs for yield potential and evaluate differences in allelic effects of QTLs on both genetic backgrounds. QTL analysis of the traits associated with lodging resistance has not been performed using reciprocal CSSLs.

In this study, we detected QTLs for lodging resistance using reciprocal CSSLs derived from a cross between Koshihikari and Takanari and identified a QTL on chromosome 1 using reciprocal NILs. This study is the first to find QTLs for the cortical fibre tissue thickness associated with culm stiffness in rice.

## Results

### Differences in breaking type lodging resistance between Koshihikari and Takanari

To detect QTLs for lodging resistance, we first dissected breaking type lodging resistance into its component traits. These traits were compared for the two parents, Koshihikari and Takanari, using the fourth and fifth internodes 15 days after heading (Table 1). Large differences in all measured parameters of both the fourth and fifth internodes were observed between Koshihikari and Takanari. The M of Takanari was much higher than that of Koshihikari. M can be further divided into two components, SM and BS. The SM of Takanari was larger than that of Koshihikari. By contrast, the BS of Takanari was lower than that of Koshihikari. These observations indicate that Takanari has a thicker and more fragile culm than Koshihikari.

### Differences in the breaking type lodging resistance among reciprocal CSSLs

As the next step in detecting QTLs for lodging resistance, we compared the component traits associated with breaking type lodging resistance in reciprocal CSSLs and the genetic backgrounds of both varieties.

**Figure 1:**
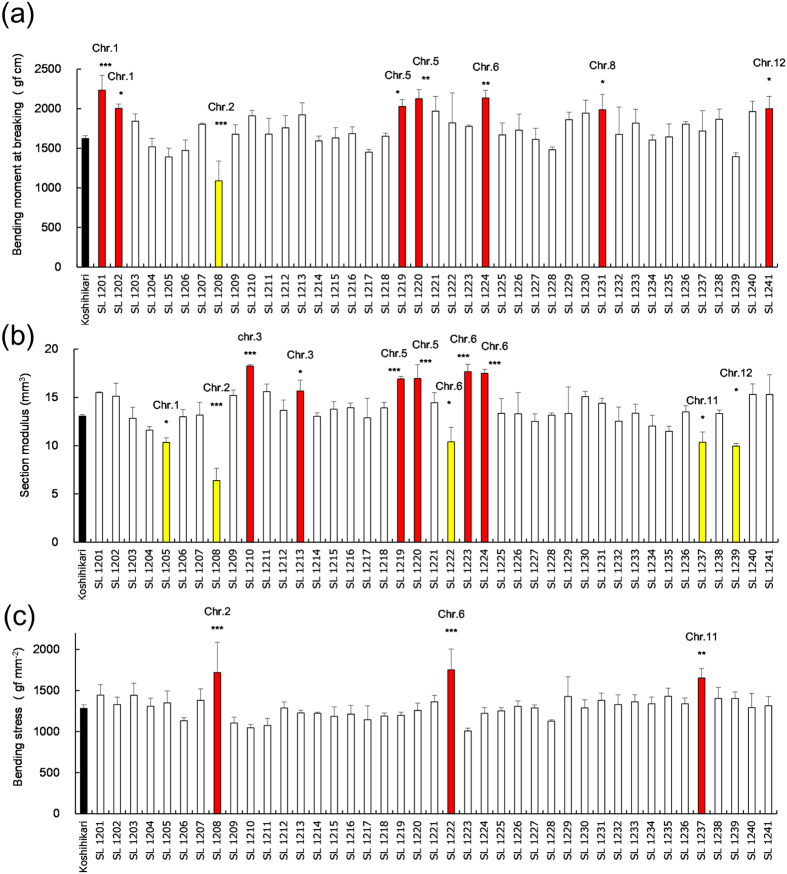
Bending moment at breaking, section modulus and bending stress in K-CSSLs. (**a**) Comparison of the bending moment at breaking of the fifth internodes 15 days after heading in 2012. (**b**) Section modulus of the fifth internodes. (**c**) Bending stress of the fifth internodes. ***P < 0.001, **P < 0.01, *P < 0.05 versus Koshihikari (Dunnett’s multiple comparison test, three replicates).

The M, SM and BS of the basal fifth internode were first compared for Koshihikari and 41 Takanari CSSLs on the Koshihikari genetic background (K-CSSLs) (Fig. 1). M was 1623 gf cm in Koshihikari (Fig. 1a) and ranged from 1089 to 2234 gf cm among the 41 K-CSSLs. Significant differences in M were detected between 8 K-CSSLs and Koshihikari. M was smaller in line SL 1208 than Koshihikari but larger in lines SL 1201, 1202, 1219, 1220, 1224, 1231 and 1241 (Fig. 1a). SM was approximately 13 mm^3^ in Koshihikari (Fig. 1b and Supplementary Fig. S1a) and ranged from 6.4 to 18.3 mm^3^ among the 41 K-CSSLs. Significant differences in SM were detected between 11 K-CSSLs and Koshihikari. SM was smaller in lines SL 1205, 1208, 1222, 1237 and 1239 (Fig. 1b and Supplementary Fig. S1b, c, h, k, l) than in Koshihikari but larger in lines SL 1210, 1213, 1219, 1220, 1223 and 1224 (Fig. 1b and Supplementary Fig. S1d–g,i,j). BS was 1282 gf mm^−2^ in Koshihikari (Fig. 1c) and ranged from 1006 to 1752 gf mm^−2^ among the 41 K-CSSLs. Significant differences in BS were detected between 3 K-CSSLs and Koshihikari. BS was not significantly smaller in these lines than in Koshihikari but was larger in lines SL 1208, 1222 and 1237 (Fig. 1c).

**Figure 2:**
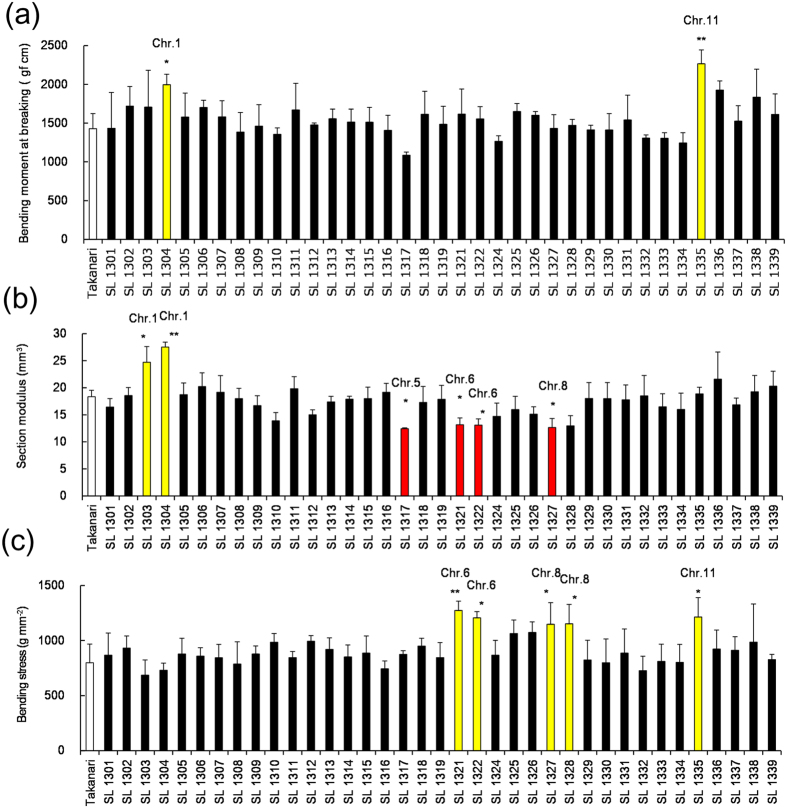
Bending moment at breaking, section modulus and bending stress in T-CSSLs. (**a**) Comparison of the bending moment at breaking of the fourth internodes 15 days after heading in 2013. (**b**) Section modulus. (**c**) Bending stress. ***P < 0.001, **P < 0.01, *P < 0.05 versus Takanari (Dunnett’s multiple comparison test, three replicates).

Next, the M, SM and BS of the basal fourth internode were compared between Takanari and 37 Koshihikari CSSLs in the Takanari genetic background (T-CSSLs) (Fig. 2). M was 1428 gf cm in Takanari (Fig. 2a) and ranged from 1086 to 2266 gf cm among the 37 T-CSSLs. Significant differences in M were detected between 2 T-CSSLs and Takanari. M was larger in SL 1304 and 1335 than in Takanari (Fig. 2a). SM was 18.4 mm^3^ in Takanari (Fig. 2b) and ranged from 12.4 to 27.5 mm^3^ among the 37 T-CSSLs. Significant differences in SM were detected between 6 T-CSSLs and Takanari. SM was smaller in SL 1317, 1321, 1322 and 1327 than in Takanari but larger in lines SL 1303 and 1304 (Fig. 2b). By contrast, BS was 799 gf mm^−2^ in Takanari (Fig. 2c) and ranged from 686 to 1274 gf mm^−2^ among the 37 T-CSSLs. Significant differences in BS were found between 5 T-CSSLs and Takanari. In SL 1321, 1322, 1327, 1328 and 1335, BS was larger than in Takanari (Fig. 2c).

### Cortical fibre tissue thickness in Koshihikari, Takanari and reciprocal CSSLs

Cortical fibre tissue thickness is a key component trait associated with culm stiffness. Koshihikari has a thick cortical fibre tissue. By contrast, the cortical fibre tissue in Takanari is very thin, which is one reason why Takanari has a more fragile culm than Koshihikari. To detect QTLs for cortical fibre tissue thickness, we compared the cortical fibre tissue thickness among the reciprocal CSSLs, Koshihikari and Takanari.

**Figure 3:**
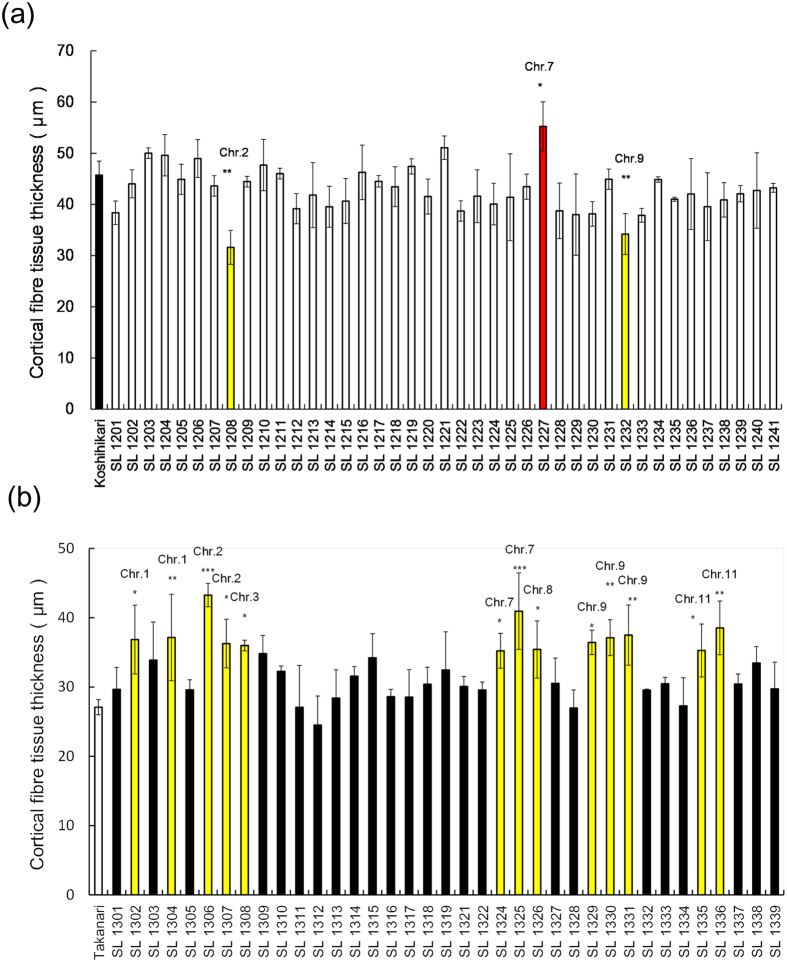
Cortical fibre tissue thickness in reciprocal CSSLs. (**a**) Comparison of the cortical fibre tissue thickness of the fifth internodes between Koshihikari and K-CSSLs 15 days after heading in 2014. **P < 0.01, *P < 0.05 versus Koshihikari. (**b**) Comparison of the cortical fibre tissue thickness of the fourth internodes between Takanari and T-CSSLs at 15 days after heading in 2014. ***P < 0.001, **P < 0.01, *P < 0.05 versus Takanari (Dunnett’s multiple comparison test, three replicates).

**Figure 4:**
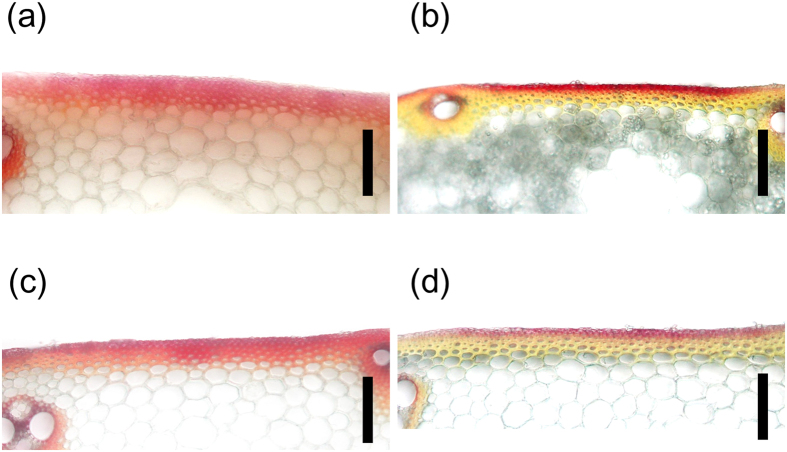
Cortical fibre tissue in K-CSSLs. Transverse sections of the fifth internodes at 15 days after heading were stained with phloroglucinol. Phloroglucinol staining (red colour) was observed in cortical fibre tissue and vascular bundles. (**a**) Koshihikari, (**b**) SL 1208, (**c**) SL 1227, (**d**) SL 1232. Scale bar: 100 μm.

**Figure 5:**
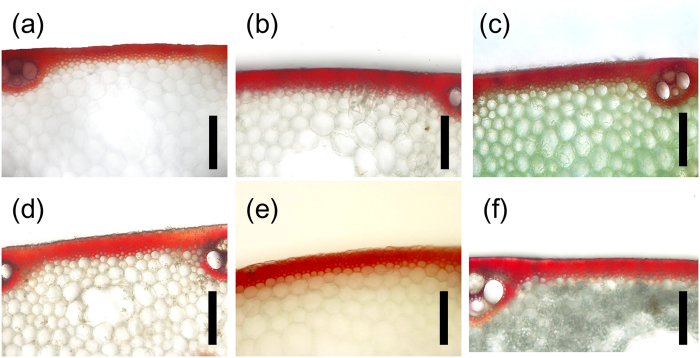
Cortical fibre tissue in T-CSSLs. Transverse sections of the fourth internodes 20 days after heading were stained with phloroglucinol. Phloroglucinol staining (red colour) was observed in cortical fibre tissue and vascular bundles. A: Takanari, B: STK-4, C: STK-6, D: STK-25, E: TK-30, F: STK-31, G: STK-36. Scale bar: 100 μm.

The cortical fibre tissue thickness was compared between Koshihikari and 41 K-CSSLs (Fig. 3a) and between Takanari and 37 T–CSSLs (Fig. 3b). The cortical fibre tissue thickness in Koshihikari was 45.7 μm (Fig. 3a) and ranged from 31.6 to 55.2 μm among the 41 K-CSSLs. There were significant differences in the cortical fibre tissue thickness between 3 K-CSSLs and Koshihikari. In lines SL 1208 and 1232, the cortical fibre tissue was thinner than in Koshihikari (Fig. 3a and Fig. 4b,d), but was thicker in lines SL 1227 (Figs 3a and 4c). By contrast, the cortical fibre tissue thickness in Takanari was 27.1 μm and ranged from 24.5 to 43.3 μm among the 37 T-CSSLs. Significant differences in the cortical fibre tissue thickness were detected between 13 T-CSSLs and Takanari. The cortical fibre tissue was thicker in lines SL 1302, 1304, 1306, 1307, 1308, 1324, 1325, 1326, 1329, 1330, 1331, 1335 and 1336 than in Takanari (Fig. 3b and Fig. 5).

### Substitution mapping of QTLs for component traits of lodging resistance

Based on the genotypes of the lines showing statistically significant differences, we mapped QTLs for component traits of lodging resistance. In addition, to estimate effective QTLs that were not dependent on genetic background, we compared the estimated candidate regions of these QTLs in the reciprocal CSSLs.

**Figure 6:**
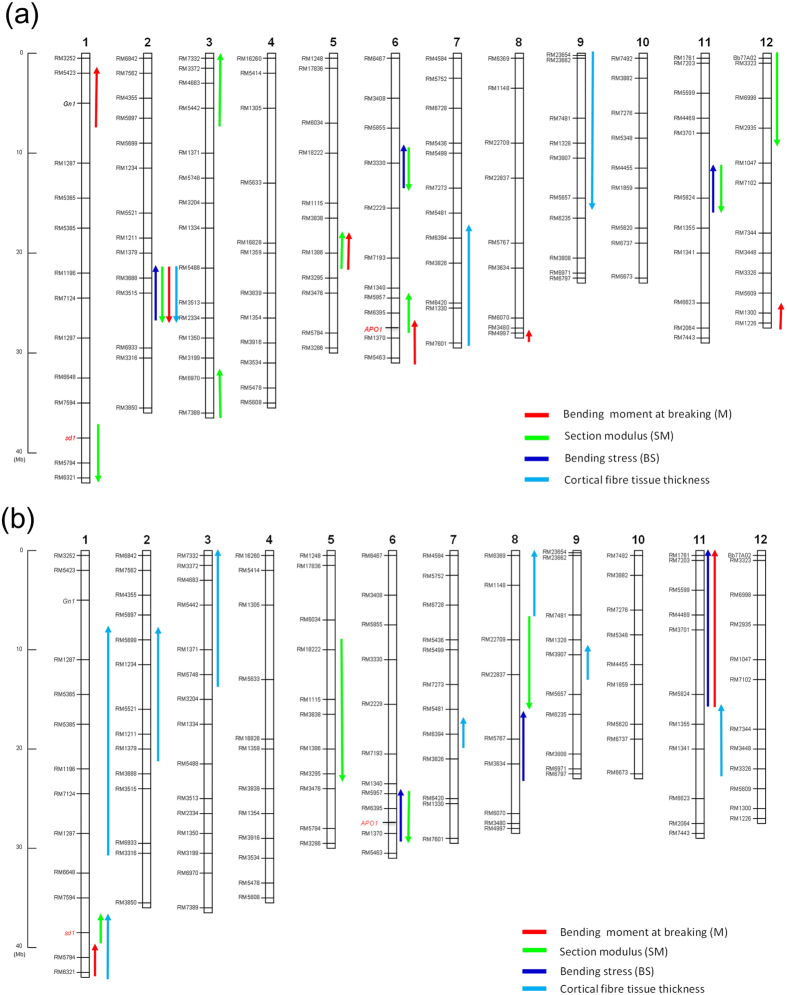
QTLs associated with lodging resistance estimated from reciprocal CSSLs. (**a**) QTLs from K-CSSLs. (**b**) QTLs from T-CSSLs. The vertical bars numbered chr.1-chr.12 denote the 12 rice chromosomes; the names of SSR markers are indicated on the left side of each bar. Coloured bars denote estimated QTLs for lodging resistance. Red: bending moment at breaking, green: section modulus, sky blue: bending stress, dark blue: cortical fibre tissue thickness. Upper arrows indicate positive effects. Lower arrows indicate negative effects.

Figure 6a shows the estimation of chromosomal locations of QTLs for component traits of lodging resistance using K-CSSLs. Substitution mapping confined QTLs for M into five regions on chromosomes 1, 5, 6, 8 and 12 where the Takanari alleles have positive effects and one region on chromosome 2 where the Takanari allele has a negative effect. For SM, we detected three regions on chromosomes 3, 5 and 6 where the Takanari alleles contributed to an increase in SM and five regions on chromosomes 1, 2, 6, 11 and 12 where the Takanari alleles contributed to a decrease in SM. For BS, we detected three regions on chromosomes 2, 6 and 11 where the Takanari alleles have a positive effect.

Figure 6b shows the estimation of chromosomal locations of QTLs for lodging resistance using T-CSSLs. For M, we detected regions on chromosomes 1 and 11 where the Koshihikari alleles have a positive effect. For SM, we detected a region on chromosome 1 where the Koshihikari alleles have a positive effect and three regions on chromosomes 5, 6 and 8 where the Koshihikari alleles have a negative effect. For BS, we detected three regions on chromosomes 6, 8 and 11 where the Koshihikari alleles have a positive effect.

Based on these results, QTLs for SM were estimated in reciprocal regions from RM7594 to RM5794 on chromosome 1, from RM3838 to RM3295 on chromosome 5 and from RM5957 to RM1370 on chromosome 6 (Fig. 6).

### Substitution mapping of QTLs for the trait responsible for culm stiffness

Next, we mapped QTLs for the cortical fibre tissue thickness, one of the traits responsible for culm thickness.

Using K-CSSLs, substitution mapping confined QTLs for the cortical fibre tissue thickness into two regions on chromosomes 2 and 9 where the Takanari alleles have negative effects. We also detected one region on chromosome 7 where the Takanari allele has a positive effect (Fig. 6a). Using T-CSSLs, substitution mapping confined QTLs for the cortical fibre tissue thickness into seven regions on chromosomes 1, 2, 3, 7, 8, 9 and 11 where the Koshihikari alleles have positive effects (Fig. 6b). From this mapping, QTLs for the cortical fibre tissue thickness were estimated in reciprocal regions from RM3907 to RM5657 on chromosome 9 and close regions near RM1379 on chromosome 2.

**Figure 7:**
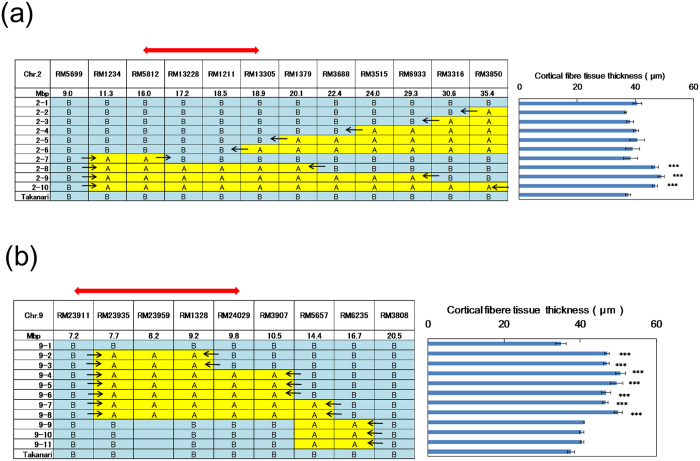
Mapping and narrowing of the QTLs on chromosomes 2 and 9 associated with cortical fibre tissue thickness using the Koshihikari segment substitution lines on the Takanari genetic background in 2015. (**a**) Chromosome 2. Red arrow indicates the candidate region (2.9 Mbp) of the QTL estimated from the phenotype data for cortical fibre tissue thickness. (**b**) Chromosome 9. Red arrow indicates the candidate region (2.6 Mbp) of the QTL estimated from the phenotype data for cortical fibre tissue thickness. A: Koshihikari genotype, B: Takanari genotype.

To identify the candidate regions for the QTL for the cortical fibre tissue thickness on chromosomes 2 and 9, substitution lines on the Takanari genetic background were developed. Using these lines, the candidate region on chromosome 2 was narrowed down to the region from RM5812 to RM 13305 (2.9 Mbp) (Fig. 7a). This region was located near the identified region of *brittle culm 5* (*bc5*)^20^. To clarify the differences in physiological function between our estimated responsible gene and *bc5*, their structural properties were compared between the substitution line 2–8 and Takanari and between *bc5* and wild type IR36 (Supplementary Fig. S2). There was no difference in the number of cell layers in the cortical fibre tissue between *bc5* and IR36 (Supplementary Fig. S2c,d,e and f), whereas a large difference was observed between substitution line 2–8 and Takanari (Supplementary Fig. S2a,b). On chromosome 9, the candidate region was narrowed down to the region from RM23911 to RM24029 (2.6 Mbp) (Fig. 7b).

### Genomic region containing *SD1*/*sd1* on chromosome 1 controls culm thickness

**Figure 8:**
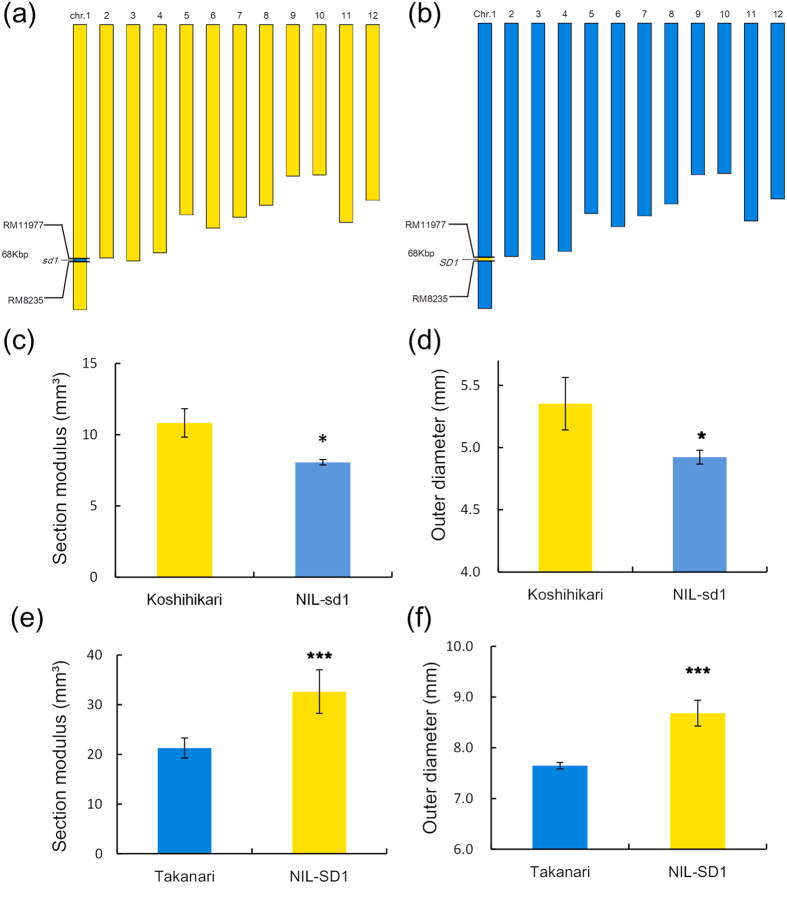
Graphical genotypes and lodging resistance of NIL-SD1/sd1. (**a**) Graphical genotype of NIL-sd1 on rice chromosome. (**b**) Graphical genotype of NIL-SD1 on rice chromosome. (**c,d**) Comparison of section modulus (**c**) and outer diameter (**d**) between Koshihikari and NIL-sd1. (**e,f**) Comparison of section modulus (**e**) and outer diameter (**f**) between Takanari and NIL-SD1. *Values are significantly different at the 0.05 level (t-test: three replicates).

A QTL for SM was estimated in a reciprocal region on chromosome 1. This region contains the *SEMI DWARF1* (*SD1*) gene; therefore, we hypothesized that the genomic region containing *SD1/sd1* is a gene controlling SM. To test the hypothesis, we developed reciprocal NILs. Figure 8a and b shows graphical representations of the genotypes of the reciprocal NILs (NIL-sd1 and NIL-SD1). NIL-sd1 has a Takanari chromosome segment substituted for a narrow region (68 kbp) on chromosome 1, including the *sd1* gene (between RM11977 and RM8235), on the Koshihikari background. By contrast, the reciprocal NIL-SD1 has a Koshihikari chromosome segment substituted for the same region, including the *SD1* gene, on the Takanari background. SM and outer diameter in NIL-sd1 were lower than in Koshihikari (Fig. 8c,d), whereas NIL-SD1 was higher than Takanari (Fig. 8e,f).

These results showed that the genomic region containing *SD1*/*sd1* contributes to the regulation of SM.

## Discussion

Several QTL and their responsive genes associated with a strong culm have been identified because this complex trait is controlled by multiple genes. In our previous studies^18^,^21^, we identified certain QTLs responsible for strong culms and culm thickness from different varieties, such as *STRONG CULM2* (*SCM2*) and *SCM3*. From Habataki, an improved high-yielding *indica* variety with strong, thick culms, we detected QTLs associated with a thick culm: *SCM1* on chromosome 1 and *SCM2* on chromosome 6. *SCM2* was identical to *APO1*, a gene reported to control panicle structure^8^. *SCM2* enhanced culm strength and increased spikelet number due to the pleiotropic effects of this gene. From Chugoku 117, a tropical *japonica* type high-yielding line with strong, thick culms, we also detected QTLs associated with a thick culm: *SCM3* on chromosome 3 and *SCM4* on chromosome 2. *SCM3* was identified as identical to rice *FINE CULM1* (*FC1*), a gene reported to positively control strigolactone signalling^21^,^22^. In Japan, Takanari is the highest yielding rice variety with superior lodging resistance^19^. To precisely identify new QTLs for lodging resistance, we detected some QTLs for the component traits associated with a strong culm using reciprocal CSSLs derived from a cross between Koshihikari and Takanari.

QTLs for section modulus were detected at the same genomic regions on chromosomes 1, 5 and 6 in reciprocal genetic backgrounds (Fig. 6). The Takanari alleles on chromosomes 5 and 6 contributed to increases in section modulus and bending moment at breaking, while the Koshihikari allele on chromosome 1 contributed to an increase in section modulus. These effective QTLs controlled section modulus by affecting culm diameter and thus enhanced the bending moment at breaking. As mentioned above, the QTL for section modulus and culm diameter on the long arm region of chromosome 6 has been identified as *SCM2,* and the responsible gene was identified as *APO1*^18^. The *APO1* allele in Habataki, a sister variety derived from the same parents as Takanari, leads to a large gain-of-function activity. The Takanari allele is also regarded as *APO1. APO1* has pleiotropic effects on the morphological features and morphogenesis of the panicle and culm. Takai *et al*.^19^, who developed reciprocal CSSLs in a cross between Koshihikari and Takanari, detected a QTL for spikelet number per panicle on the long arm of chromosome 6 in both reciprocal genetic backgrounds. It was confirmed that reciprocal CSSLs are useful materials for the precise estimation of effective QTLs for agronomically important traits.

Our detected QTLs for SM on the 68-kbp long arm region of chromosome 1 contained the *SD1* gene (Fig. 6). We found 5 candidate genes using RAP-DB, including *sd1* (Supplementary Table S1). Takanari has the same *sd1* allele inherited from Dee-geo-woo-gen and IR8^19^,^23^. The *sd1* Takanari allele contains a 383-bp deletion, which induces a frameshift^18^,^22^, whereas Koshihikari, which has a long culm, has the *SD1* allele. *SD1* encodes a physiologically active gibberellin biosynthetic enzyme, GA20 oxidase^4^,^5^. NIL-sd1 had a significantly reduced SM and outer culm diameter. By contrast, NIL-SD1 had an increased SM and outer culm diameter. Okuno *et al*.^24^ showed that GA regulates the culm diameter using *sd1* mutants, and there is a positive correlation between GA and culm thickness in rice based on observations of GA-deficient mutants and a high-GA producing line. GA promotes not only axial cell elongation but also radial cell division^25^. The GA level might affect the enlargement of culm diameter by increasing the number of parenchymal cells in the culm. In this study, we also found that Koshihikari, which has long and fine culms, not only had fine culm genes but also thick culm genes in the genomic region including *SD1* on chromosome 1. Further studies are needed to confirm whether *SD1*/*sd1* regulates culm thickness in this genomic region on chromosome 1.

The bending stress in Takanari was smaller than in Koshihikari (Table 1). In our previous study^11^, the bending stress in the typical Japanese *japonica* fine-culm varieties was higher than in the typical tropical *japonica* and *indica* thick-culm varieties. No QTLs for bending stress were detected in the same genomic regions in reciprocal genetic backgrounds (Fig. 6). Bending stress is a complex trait controlled by many responsible traits^12^. This fact might be why QTLs could not be detected in the same regions reciprocally and why these regions did not overlap with QTLs for the responsible traits in the same regions independently.

We attempted to dissect bending stress into responsible traits, such as the development of cortical fibre tissue in internodes and the accumulation of cell wall components. In this study, Koshihikari had thicker cortical fibre tissue than Takanari. QTLs for cortical fibre tissue thickness were detected in the genomic regions of chromosomes 2 and 9 on both genetic backgrounds using reciprocal CSSLs (Fig. 6). This study is the first to identify QTLs for cortical fibre tissue thickness in rice. The candidate regions on chromosomes 2 and 9 were narrowed down to within 3 Mbp on the Takanari genetic background (Fig. 7). The narrowed region on chromosome 9 was identified as the same region estimated by reciprocal CSSLs (Fig. 7b), whereas the narrowed region on chromosome 2 was slightly shifted to the short arm (Fig. 7a). On chromosome 2, *brittle culm5* (*bc5*) is located in a region near our detected region^20^. The responsible gene for *bc5* has not yet been identified. It has been reported that in *bc* mutants, the accumulation of cell wall components and the thickness of the secondary walls in the sclerenchyma tissue of node are reduced^25^,^26^. We compared the differences in cortical fibre tissue thickness between the *bc5* mutant and wild type IR36. We found that there was no difference in this internode thickness between the *bc5* mutant and IR36, whereas a large difference was found between the substitution line 2–8 and Takanari (Supplementary Fig. S2). Furthermore, the brittle culm phenotype of *bc5* was obvious in nodes but not in internodes^20^. Further studies are needed to identify the genes responsible for the QTL for cortical fibre tissue thickness and *bc5* on chromosome 2.

Responsible genes associated with the biosynthesis of cellulose or hemicellulose have been isolated^26^,^27^,^28^,^29^,^30^,^31^. However, QTLs and genes responsible for the development of cortical fibre tissue have not yet been identified. Physiological factors associated with the differentiation and development of cortical fibre tissues in rice internodes are also unknown. Therefore, it is necessary to identify QTLs for the development of cortical fibre tissue and to isolate the responsible genes.

Some QTLs for strong culm traits were detected in only one of the two genetic backgrounds. On chromosome 3, the most effective QTL for section modulus was detected only in the Koshihikari genetic background (Fig. 6). This result indicates that this locus in Takanari may be controlled by epistatic interactions with other genes in the Koshihikari genetic background. QTLs for the cortical fibre tissue thickness, indicating that the Koshihikari allele has a positive effect, were detected on chromosomes 1, 3, 8 and 11 only in the Koshihikari genetic background (Fig. 3). On chromosome 7, QTLs for the cortical fibre tissue thickness with a positive effect were detected in both CSSLs, regardless of their genetic backgrounds. Different epistatic genes might affect the expression of these phenotypes in both genetic backgrounds, independently. We could not identify the epistatic genes using only CSSLs^17^. Further studies are necessary to identify QTLs controlled by epistatic genes using other genetically segregated populations.

In this study, we detected major QTLs associated with strong culm traits, such as culm thickness and culm stiffness, using a set of reciprocal CSSLs for precise estimation. We suggest that lodging resistance in the *indica* variety Takanari could be improved by introducing the allele of the *japonica* variety Koshihikari for thick cortical fibre tissue and that lodging resistance in Koshihikari could be improved by introducing the Takanari allele for a thicker culm outer diameter. To improve the lodging resistance of these varieties, we must further research the genetic diversification of these alleles in rice and identify the important QTLs and their responsible genes for lodging resistance.

## Materials and Methods

### Plant materials

The rice (*Oryza sativa* L.) cultivars Koshihikari and Takanari, 37 Takanari CSSLs on the Koshihikari genetic background (T-CSSLs, SL 1201-1238) and 41 Koshihikari CSSLs on the Takanari genetic background (K-CSSLs, SL 1301-1341) were analysed in 2012 and 2013 (Supplementary Table S1). To narrow down the candidate regions of QTLs on chromosomes 1, 2 and 9, recombinant homozygous lines on chromosomes 1, 2 and 9 were evaluated in 2014 and 2015, and the *bc5* mutant line and its wild type IR36 were analysed in 2015.

### Cultivation

Details of cultivation were similar in each of the four years from 2012 to 2015. Seeds were sown in nursery boxes on May 8, 2012. Seedlings were transplanted on May 31 to a paddy field of the University Farm in Tokyo on alluvial soil of the Tama River at a rate of one plant per hill. The planting density was 22.2 hills m^−2^, with a spacing of 15 cm × 30 cm. As a basal dressing, compound fertilizer was applied at a rate of 5.0 kg 10 a^−1^ for N and 6.0 kg 10 a^−1^ for P_2_O_5_ and K_2_O.

### QTL detection using reciprocal CSSLs and mapping of detected QTLs.

Using K-CSSLs and T-CSSLs, QTLs for strong culm traits associated with lodging resistance were detected. A QTL was considered to be present when the average of three replicates was significantly different between a CSSL and the recurrent parent.

To map the detected QTLs for cortical fibre tissue thickness on chromosomes 2 and 9, a BC_1_F_2_ population was derived from a cross between Takanari and its CSSL carrying the QTL region. Recombinant homozygous plants (BC_1_F_4_) were selected from the self progenies of BC_1_F_3_ through MAS and were used to define the QTL region. The phenotypes of the recombinant homozygous lines were compared with Takanari.

Similarly, NILs carrying *SD1*/*sd1* were selected using MAS and were used to evaluate the reciprocal effects of the QTL for SM. Genomic DNA was extracted from leaves using the cetyltrimethylammonium bromide (CTAB) method^32^, and pure DNA samples were used to genotype single-sequence repeat markers^33^,^34^.

### Measurements of culm strength

Physical parameters of culm strength were used for precise phenotyping of breaking type lodging resistance. Thirty main culms were sampled from each plot, and eight stems were selected with average culm length and basal internode length. For the comparison between both parents, we measured the culm strength of the fourth and fifth internodes in the same main culm as the independent experiment. For reciprocal CSSLs, the culm strength could not compare at the same internodes between K-CSSLs and T-CSSLs. The culm strength of the fifth internode was compared between K-CSSLs and Koshihikari, because the almost lines had the elongated fifth internode, and the culm strength of the fourth internode was compared between T-CSSLs and Takanari, because many lines did not elongate the fifth internode. Thus, the culm strength was measured at the different internodes in K-CSSLs and T-CSSLs, independently.

The bending load at breaking was measured at a distance of 4 cm between supports by the method of Ookawa *et al*.^11^ using a Tensilon RTG-120 universal testing machine (A&D, Tokyo, Japan), which is a useful tool for the precise evaluation of lodging resistance traits in rice.

Physical parameters were calculated by the following formula:

Bending moment at breaking = Section modulus × Bending stress (gf.cm)

Section modulus = π/32 × (a_1_^3^b_1_-a_2_^3^b_2_)/a_1_

where a1 is the outer diameter of the minor axis in an oval cross-section, b1 is the outer diameter of the major axis in an oval cross-section, a2 is the inner diameter of the minor axis in an oval cross-section, and b2 is the inner diameter of the major axis in an oval cross-section.

Bending stress is a mechanical parameter for lodging resistance that is influenced by the chemical composition of the culm, such as its cellulose and lignin content.

### Observation of transverse sections of basal internodes

The fourth or fifth internodes were sampled 15 or 20 days after heading and fixed in FAA. For lignin histochemical staining, transverse sections of internodes were stained with phloroglucinol solution. Internode sections were observed with a MSX-500Di digital microscope (MORITEX, Saitama, Japan), and the cortical fibre tissue thickness was measured using SCM Measure software (MORITEX, Tokyo, Japan). The cortical fibre development was observed using a TM3000 scanning electron microscope (Hitachi High Technologies, Tokyo, Japan).

### Statistical analysis

Statistical comparison of multiple sets of data was carried out using Dunnett’s multiple comparison test and *t*-test.

## Additional Information

**How to cite this article**: Ookawa, T. *et al*. Precise estimation of genomic regions controlling lodging resistance using a set of reciprocal chromosome segment substitution lines in rice. *Sci. Rep.* **6**, 30572; doi: 10.1038/srep30572 (2016).

## Supplementary Material

Supplementary Information

## Figures and Tables

**Table 1:** Breaking type lodging resistance in Koshihikari and Takanari.

Variety	Bending moment at breaking		Section modulus		Bending stress	
	gf cm		mm^3^		gf mm^−2^	
	IV	V	IV	V	IV	V
Koshihikari	1209	1413	9.8	10.6	1261	1371
Takanari	1848	2266	22.7	23.3	828	979
	*	**	*	**	*	*

The fourth and fifth internodes of main culms were sampled at 15 days after heading in Koshihikari and Takanari in 2012.

* and **, Values significantly different at the 0.05 and 0.01 levels, respectively (t-test: three replicates).
