# Supplementary material for: Precise estimation of genomic regions controlling lodging resistance using a set of reciprocal chromosome segment substitution lines in rice
Source: Sci Rep. 2016 Jul 28;6:30572. doi: 10.1038/srep30572 (PMC4964586; doi:10.1038/srep30572)
Supplement: Supplementary Information [file srep30572-s1.pdf]

Supplementary Information

Precise estimation of quantitative trait loci controlling lodging resistance using a set of reciprocal chromosome segment substitution lines in rice

Taiichiro Ookawa[1*], Ryo Aoba[1], Toshio Yamamoto[2], Tadamasa Ueda[2], Toshiyuki Takai[3], Shuichi Fukuoka[2], Tsuyu Ando[2], Shunsuke Adachi[1], Makoto Matsuoka[4], Takeshi Ebitani[5], Yoichiro Kato[6], Indria Wahyu Mulsanti[1], Masahiro Kishii[7], Matthew Reynolds[7], Francisco Piñera[7], Toshihisa Kotake[8], Shinji Kawasaki[9], Takashi Motobayashi[1] and Tadashi Hirasawa[1]

[1]Institute of Agriculture, Graduate School, Tokyo University of Agriculture and Technology, Fuchu, Tokyo 183-8509, Japan, [2]NARO Agrogenomics Research Center, Tsukuba, Ibaraki 305-8602, Japan, [3]Japan International Research Center for Agricultural Sciences, Tsukuba, Ibaraki 305-8686, Japan, [4]Bioscience and Biotechnology Center, Nagoya University, Nagoya, Aichi 464-8601, Japan, [5]Agricultural Research Institute, Toyama Agricultural, Forestry & Fisheries Research Center, Toyama, Toyama 939-8153, Japan, [6]International Rice Research Instituite, Los Banos, Phillipines, [7]International Maize and Wheat Improvement Center, Texcoco, 56237, Mexico, [8]Graduate School of Science and Engineering, Saitama University, Saitama 338-8570, Japan, [9]Department of Plant Physiology, National Institute of Agrobiological Sciences, Tsukuba, Ibaraki 305-8602, Japan

Correspondence and requests for materials should be addressed to T.O. (ookawa@cc.tuat.ac.jp)

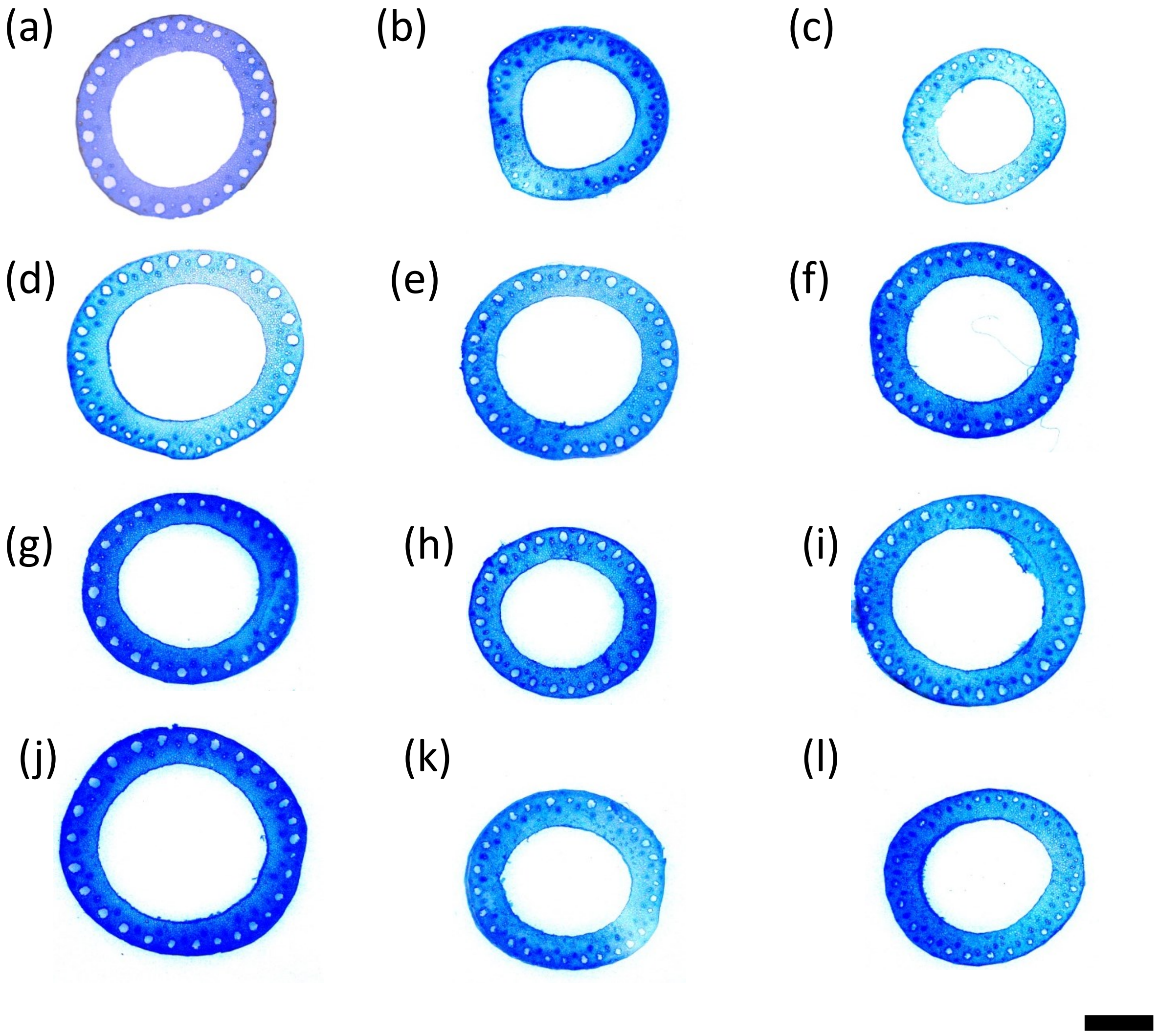

Supplementary Figure S1 Cross sections of the fifth internodes in K-CSSLs. (a)Koshihikari, (b) SL 1205, (c) SL 1208, (d) SL1210, (e) SL 1213, (f) SL-1219, (g) SL 1220, (h) SL 1222, (i) SL1223, (j) SL 1224, (k) SL 1237, (l) SL 1239. Scale bar:2mm.

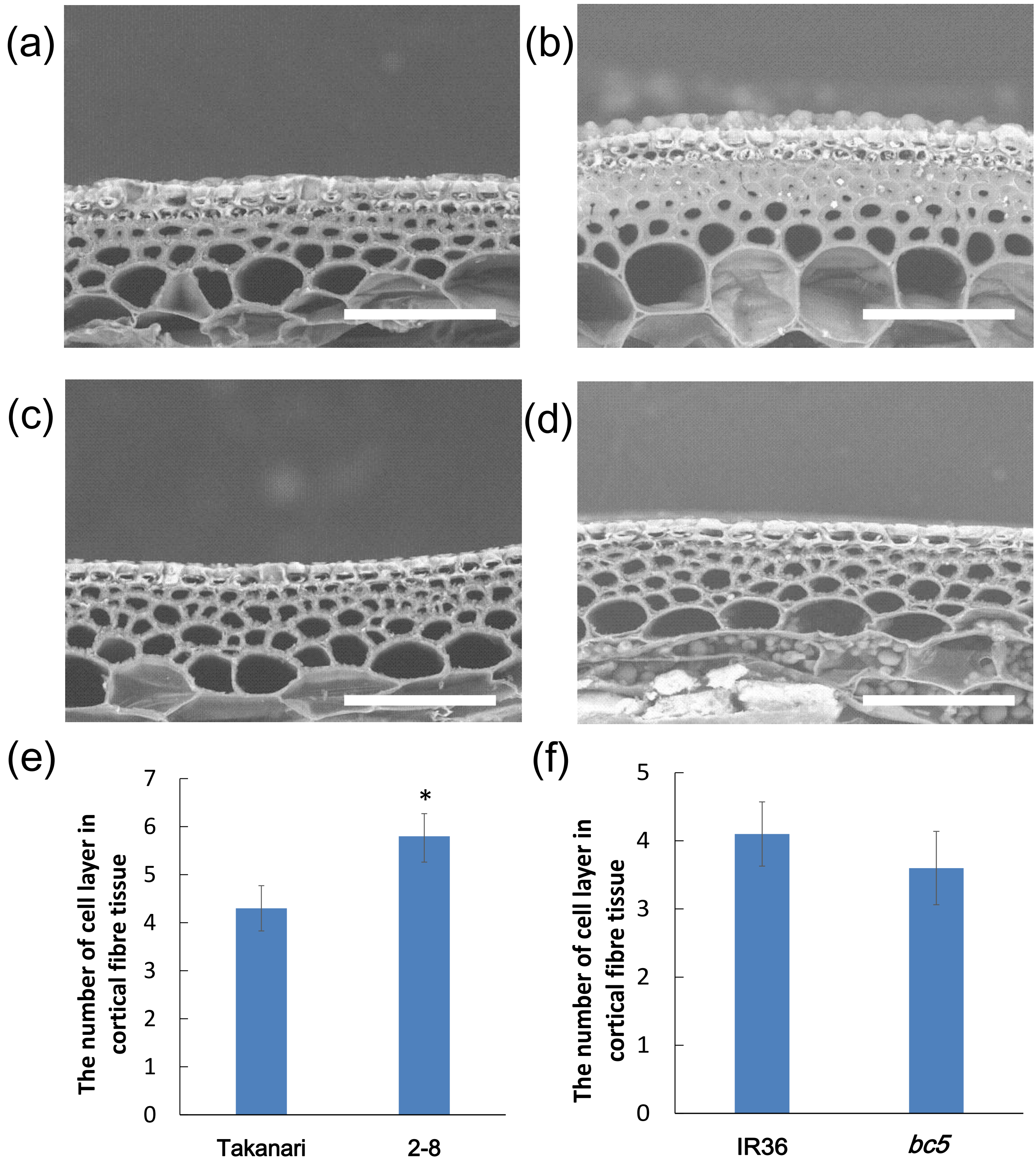

Supplementary Figure S2 Diifferences in the development of cortical fibre tissue between Takanari and 2-8 and between IR36 and *bc5*. A-B:Transverse section of the fourth internodes at 15 days after heading were observed with scanning microscope. (a) Takanari, (b)2-8, (c) IR36, (d) *bc5*. Scale bar:50μm. (e)-(f): The number of cell layer in cortical fibre tissue, (e) Takanari and 2-8, (f) IIR36 (wild type) and *bc5*. * indicates statistically significant difference at the 0.05 level (t-test: three replicates).

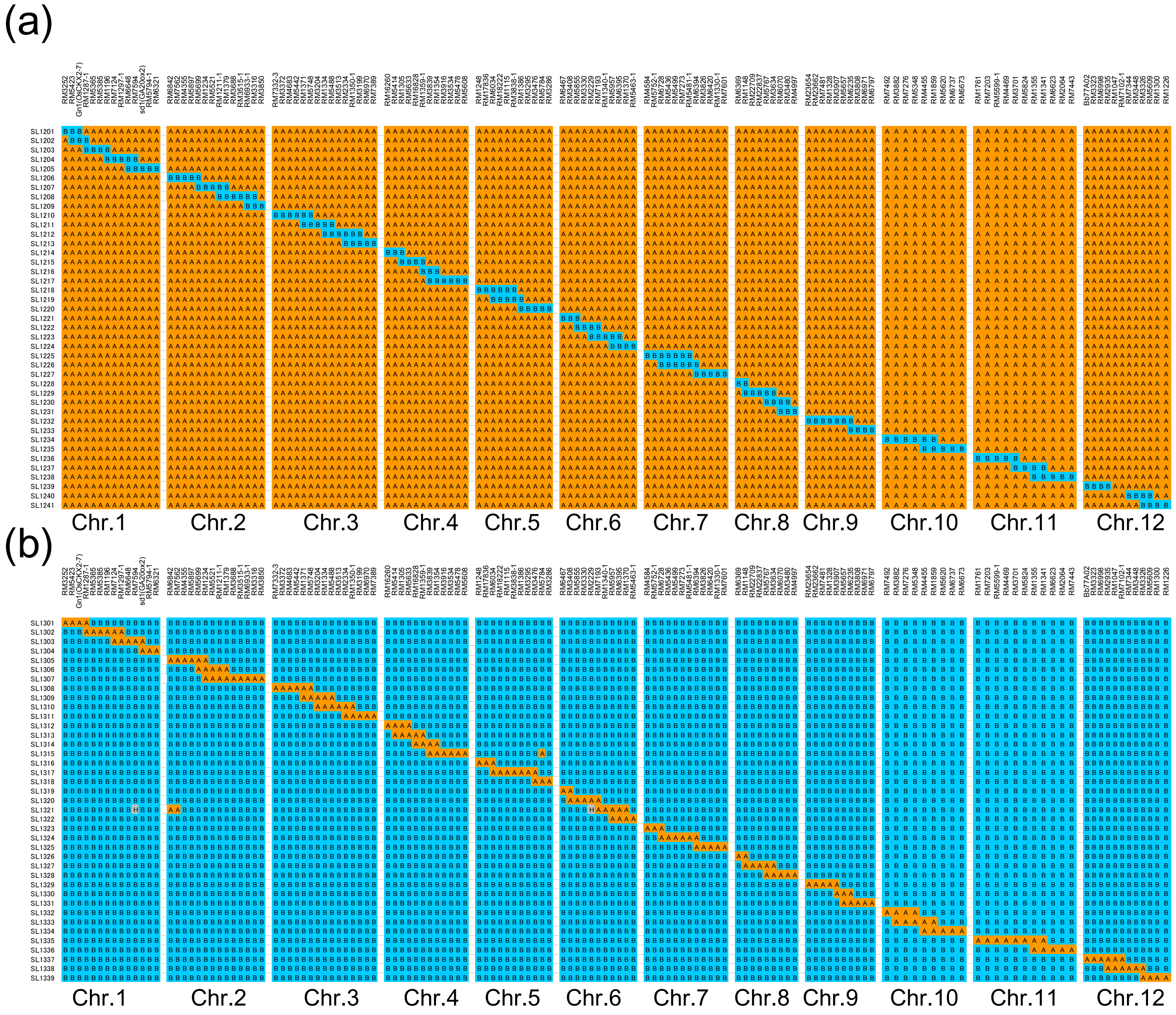

Supplementary Figure S3 Graphical genotypes of the reciprocal CSSLs. (a) 41 K-CSSLs, (b) 39 T-CSSLs. Orange regions indicate homozygosity for Koshihikari; blue regions indicate homozygosity for Takanari. Gray region indicates heterozygosity. Genotypes of the 141 SSR markers in both CSSLs are shown in the upper parts of graphs. A: Koshihikari genotype, B: Takanari genotype.

Supplementary Table S1 Candidate genes on the 68kbp genomic region on chromosome 1.

Gene	Description	Position
Os01g0883800	GA 20-oxidase2, GA metabolism	chr01:38382385..38385469 (+ strand)
Os01g0883900	Protein of unknown function DUF248, methyltransferase putative family protein	chr01:38386425..38391652 (- strand)
Os01g0884300	No apical meristem (NAM) protein domain containing protein.	chr01:38398996..38401481 (- strand)
Os01g0884400	Armadillo domain containing protein	chr01:38409242..38413057 (+ strand)
Os01g0884500	Plus-3 domain containing protein	chr01:38413181..38424557 (- strand)
